# Faces of depression. Why do we need Batman, Joker, and Bane?

**DOI:** 10.3389/fpsyg.2025.1553269

**Published:** 2025-08-11

**Authors:** Simona Reghintovschi, Anatol Eduard Reghintovschi

**Affiliations:** Faculty of Psychology, Titu Maiorescu University, Bucharest, Romania

**Keywords:** superheroes, Batman, Joker, Bane, Dark Knight, Bruce Wayne, depression, film psychoanalysis

## Abstract

Since 1939, when they first appeared in comic books, Batman and his antagonists have fascinated spectators with their endless drama. How can we understand the longevity of these characters in popular culture and the enthusiasm of the audience? Using a qualitative methodology informed by object relations theory and film analysis, the authors indicate that the drama enfolding on the cinema screen mirrors the depressive dynamics taking place in the inner word of the viewer who, by watching the movie, can master or, at least, keep at bay the terrifying conflicts between fragments of his ego represented by Batman, Joker, and Bane. In Gotham City, like in everyone's inner personality, the human conflicts related to dependency, loss, mourning, and change are always present.

## 1 Introduction

In the last few decades, our worlds, all of them, are passing points of no return at a pace exceeding the level of climate change. New forms of distress and mental suffering are emerging, next to the old ones (Luyten and Fonagy, 2021, p. 248). Nowadays, depression is estimated to be the second leading cause of disease burden worldwide by 2030 (Moussavi et al., 2007; World Health Organization, 2018), becoming “the signature of our time” (Leuzinger-Bohleber, 2024, p. 22).

Social reality undergoes chaotic and extreme developments. Profound instability, unpredictability, and loss can be a source for those states of mind, when one “is unconsciously and firmly convinced that nobody but nobody can help him in a situation of extreme, life-threatening danger and dehumanization, but that he is totally left alone and completely incapable of freeing himself from the unbearable situation” (Leuzinger-Bohleber, 2021, p. 100).

Sometimes imaginary worlds are summoned to give an answer. Our superheroes and their villains, inhabiting the worlds of comics, such as those of DC and Marvel, the worlds of fantasy and science fiction literature, and the narratives unfolding on the silver screen, offer opportunities for encounters that allow us to better understand ourselves, our relationships with others, and the world we live in. The stories illustrated in films become sources of meaning and hope, allowing a chance for personal growth (Tisseron, 2003; Izod and Dovalis, 2015; Fatemi, 2022; Hamburger, 2024). Moreover, these stories are sometimes about defeating depression, the new global villain.

The questions that guided our study are: What makes viewers go to the cinema to see the Batman Trilogy? What aspects of their personality do they recognize in the characters on screen? What internal conflicts can they find in the relationships between the characters on screen? We assume that, in Christopher Nolan's Batman trilogy, viewers can find echoes of their own depressive experiences and ways to overcome them. As Hamburger states, “cinema is not therapy, yet every great film touches us in some way as if it reminded us of something” (Hamburger, 2024, p. 227).

## 2 Methods

In this study, we will use the psychoanalytic perspective on the relationship of the viewer with the film. Although many other aspects are important—economic, social, and political—in this study, we will pay attention mainly to the psychological aspect of the viewer's interaction with the movie on screen. Our hypothesis is that the Batman trilogy illustrates the struggle with depression and the process of overcoming it. As the depressive experiences are present on a continuum from non-clinical to clinical spectrum, through identification with the characters on the screen, the spectator could recognize something from their own struggle with depressive states and find a possible way out from the labyrinth of depression.

Since Wundt's first writings about perception, we know that perception is, in fact, apperception (Schultz, 2013). Projective tests prove it again and again. Something from our mind is instilled into and colors the way we live and perceive the world in everyday life, including going to the movies. Many years ago, Murray (1938) considered that the spectators, by vicarious living, empathically participate in the action of the movies that they especially enjoy, engaging themself with the objectification by someone else of tendencies similar to their own inhibited needs.

The qualitative methodology used in this study is based on two pillars: the psychoanalytical approach of the spectator's relationship with the movie and the systematic analysis of the film proposed by Mikos (2013).

### 2.1 The spectator and the film

First, films could be seen as “modern forms of myths and fairy tales” (von Franz and Boa, 1988/1994, p. 94), replacing the storytelling from old times. Based on psychoanalytic view on fairy tales (Bettelheim, 2010; Cashdan, 2014; Róheim, 1953) we consider that, regardless of the medium used for their transmission, they face the viewer with “the basic problems of life, particularly those inherent in the struggle to achieve maturity” (Bettelheim, 2010, p. 183) and “caution against the destructive consequences if one fails to develop higher levels of responsible selfhood” (Bettelheim, 2010, p. 184). Similar to fairy tales and myths, films could be seen as using the “forgotten” symbolic language (Fromm, 1951), where inner experiences, feelings, and thoughts are conveyed as though they are tangible sensory experiences or events in the external world. This language operates on a different logic than the typical one we use in our daily interactions, prioritizing intensity of feelings and associations over time and space. It represents the unique universal language developed by humanity, consistent across all cultures and throughout history. It is “the one foreign language that each of us must learn” (Fromm, 1951, p. 10). Because, as Fromm notes, “both dreams and myths are important communications from ourselves to ourselves. If we do not understand the language in which they are written, we miss a great deal of what we know and tell ourselves in those hours when we are not busy manipulating the outside world” (Fromm, 1951, p. 10).

Through symbolic language, films place unconscious tendencies in the realm of consciousness, giving the viewer the chance to engage with and understand them, suggesting why we ought to strive for higher integration of the self, and what is involved in this process. From this perspective, analyzing the film means discovering the underlying unconscious currents that inform the structure of the movie. To this end, we add another point of view: the films as dreams.

The perspectives that consider the similarities between films and dreams and between viewers and dreamers help us understand the unconscious dimension of the interaction between the viewer and the movie. Some authors [Eberwein, 1984; Dervin, 1985; Lebovici, 1948; see also Rascaroli (2002) for a comprehensive perspective on oneiric metaphor in film theory] describe the spectator's experience in the cinema as similar to the experience of dreaming. In the cinema, in a darkened room, the images on the screen unfold before our eyes in a flow that can only be stopped by the spectator leaving the room. Often, the illustrated story does not adhere to the spatial and temporal laws that guide us in waking life in the external world. At the same time, various emotions awaken and succeed one another, as our response to what seems to be happening “really” on the screen—an experience that reminds us of our existence as dreamers. In this situation, the screen becomes an extension of ourselves” (Eberwein, 1984, p. 35).

How does this extension occur? Winnicott tells us that, intercalated between the subjective realm of experience and the realm of objective experience, there is “an intermediate area of experience,” contributed by both internal reality and external life. The role of this area is to maintain internal and external reality as distinct yet interrelated. “This intermediate area of experience, unchallenged in respect of its belonging to inner or external (shared) reality, constitutes the greater part of the infant's experience, and throughout life is retained in the intense experiencing that belongs to the arts and to religion and to imaginative living, and to creative scientific work” (Winnicott, 1971, p. 14). According to Konigsberg (1996), we tend to use film as a transitional object and “we slip into a state of half-wakefulness, into a reverie that weakens our defenses and sets loose our own fantasies and wishes to interact and fuse with the characters and even the landscape that we see on the screen” (Konigsberg, 1996, p. 885). In the front of the screen, amidst the constant unfolding of the film material, the spectators engage in a form of play in which, for a moment, they adopt, without the risk of becoming stuck there, one of the roles that the film, as a transitional phenomenon, has prepared for them, joining the hero or heroine and participating in patterns of life and adventure. Beebe (2001) argues that although the images projected on the screen are the same, each spectator will perceive them according to their predispositions and experiences. Furthermore, the experience awakened by the film continues long after the screen has gone dark. The spectator's memory of the film's content, form, and meanings falls under the pressure of their needs and personal history. The emotional impact of the moving images on the spectator is not fully determined; what holds significance for them is influenced by a subjective reading.

Moreover, in this dream-like state when “the screen becomes an extension of ourselves” (Eberwein, 1984) and the barriers between the conscious and unconscious lessen, the mechanism of projection and introjection occurs with greater force, and unconscious scenarios are activated more powerfully.

How can we understand these unconscious scenarios? We need to add another piece to the puzzle—the understanding of the inner world through the lens of object relations theory.

For Fairbairn, dreams represented “dramatizations or ‘shorts' (in the cinematographic sense) of situations existing in inner reality” (Fairbairn, 2001, p. 99), and the figures appearing in dreams represent parts of the dreamer's own personality—parts of the “ego” or internalized objects. Dreams are “pictorial representations of a phantasy” (Segal, 2005, p. 54). The dreamer is at the same time the spectator and the director of this “short movie” about his inner world based on the plot of his unconscious phantasies that “underlies and colors all [our] activities however realistic” (Segal, 2005, p. 79).

As Clarke (1994) argues, as dreamers, we are already familiar with the form of cinema spectatorship. The cinema's ability to grab and hold our attention is related to the underlying mechanism of projective identification:

“We lend or invest our emotions to the characters on the screen in a projective move, then collapse the distance between them and us through identification, so it is our own emotions that are being mobilized, our own internal world that is being dramatized, brought back to life, to participate fully with love and affection and then be tragically cast back to sleep.” (Clarke, 1994, p. 379)

We know that dreams have manifest and latent contents (Freud, 1900/1953). Manifest contents are immediately accessible, but latent contents of the dreams are covered and have to be revealed by analysis. The source of meaning can be found at the latent level. However, a question appears: who is the dreamer who “dreams” the film? The only possible answer seems to be—the movie viewer. As Sklarew (2020) mentioned, “films provide dreams that we wished for” (Sklarew, 2020, p. 402). They fulfill our fantasies and “give us the dreams we never had, the dreams we yet await” (Eberwein, 1984, p. 42). It is as if the spectator creates a “dream” from the “latent content” of the film unfolding on the screen, transforming it into a “personal film” by the inner workings of his mind.

This process is familiar to psychologists using projective techniques in personality assessment. Be it answers given to the Rorschach inkblots or imagined stories based on the TAT images, the subject constructs a response to the new life situation in which he or she is placed (to offer a response to an ambiguous stimulus) and organizes the experience by mobilizing all the resources of his/her personality, conscious and unconscious. The subject responds not only to the manifest, objective content of the images, but also to their latent content. Beyond the perceptual stimulations of the manifest content with a high degree of ambiguity, the images are structured at the phantasy level; they present a latent content that can reactivate different conflictual issues, awakening phantasmatic and affective resonances (Anzieu et al., 2017). (Anzieu et al. 2017), Chabert (2012), and many other authors have elaborated on this theme and identified the latent content in Rorschach and TAT images. In analyzing the stories offered by the subject, the correspondences between the latent content of the subject's discourse and the latent content of the material are tracked. Moreover, in understanding the meaning of the subject's story, “we undertake a kind of psychological literary criticism, seeking in the choice of language, imagery and sequence of development, as well as in the narrative detail, cues as to the story-teller's inner experience of his creative effort and his creation” (Schafer, 1958, p. 181). What interests us here is precisely this possibility to identify the latent content of an image (Shentoub and Debray, 1971), an inkblot (Chabert, 2012), a story (Duss, 1997), and, extrapolating, the latent content of a movie.

We arrive at the latent content through interpretation, or by what Schafer (1992) describes as “a sense of an answer”. Most papers dealing with the limitations of applied psychoanalysis outside the clinical realm stress the difference between a live patient and the text—the lack of free associations and the patient's response to interpretation in the applied field. Moreover, there is the risk that interpretation in applied psychoanalysis speaks most about the analyst than about the film, as countertransference fantasies could be easily projected and crystallized in interpretation.

To avoid the troublesome aspects of “pathography” and wildly “applied analysis,” we include in our methodology self-reflexivity of the analyst who is aware of his emotional reaction to the film. We propose the applied analytic attitude of the analyst as “viewer,” who encounters the movie with a free-floating emotionality, lets himself be moved by it, and observes his own emotional reactions, which can be described as transference and countertransference. Observing his emotional reactions to the movie, and understanding them, the inherent subjectivity embedded in interpretation can be brought to light through self-reflection. As Berman (2003) noted, “transference (broadly defined) and interpretation tend to intermingle, both in the clinical analytic encounter, and in any reading/viewing of art, be it by laymen, analysts or other scholars” (Berman, 2003, p. 119).

We translated the method used in the study of organizational culture (Hinshelwood and Skogstad, 2002) to the study of cultural material (Reghintovschi, 2023), and employed triangulation through group discussions. Devereux (1967) suggests that the triangulation, adding another observer to the observer–subject dyad, could provide a more complete image of an observed phenomenon. A superior objectivity of either of the two accounts by authors having different personality makeups can be obtained if they are compared in such a way that “the systematic errors of author A are detected and corrected by calibrating them against the (different) systematic errors of author B—and vice versa” (Devereux, 1967, p. 137). To reduce the effect of the researcher's bias due to affective reactions to the film, group discussions could be used. Members of the group can validate or invalidate the interpretation made based on their identification with different characters of the movies, similar to what happens in a psychoanalytic session.

Finally, we add systematic film analysis to complete the methodology used in this study. Grounding film analysis in communication and cultural studies, Mikos (2013) offers the theoretical apparatus and the methodological tools for discerning how the structural function of film text is significant for the reception. Special attention will be given to the five levels indicated by Mikos (2013): content and representation, narration and dramaturgy, characters and actors, esthetics and configuration, and context.

## 3 Case description

### 3.1 A short history of Batman

Batman (30th March 1939, Bob Kane and Bill Finger), along with a few other characters (Superman 1938, Jerry Siegel and Joe Schuster; Joker 1940, Kane, Finger, and Robinson), has a very long and successful career in the world of DC comics (then Detective Comics, National Periodical Publications); and not only there.

However, it is a bit too much to say that Batman has a history, as he is evolving from “no primary urtext” and he “has rather existed in a plethora of equally valid texts constantly appearing over more than five decades” (Uricchio and Pearson, 1991/2015, p. 207). “No history” says that any Batman, or any Joker, or…, is an inter-text and an inter-image, “constructed as a mosaic of quotations; any text is the absorption and transformation of another” (Kristeva, 1984, p. 64). And yet, he has a special type of historicity. “Three times in forty years Batman became a dominant cultural symbol in America” (Levitz, 2015/1991, p. 19), and a history of the superhero should encompass what is defined through popular memory, which “is integral to everyday experience; it is memory for the public and understood as being contingent and open rather than definitive and closed” (Spiegel and Jenkins, 2015/1991, p. 173). In this way, we can say that his history resides within the tension between how Batman is recalled and how he is expected by audiences and fans. Batman's actions are an answer to the world around him, and Gotham City is but a reflection of the world at large. Multiple narrativization of Batman, simultaneously present for audiences (Collins, 1991, p. 153), as well as the absence of an urtext, makes Batman a protean character, suited for projection as any palimpsest is, in the end, open rather than definitive.

So, Batman's history, a loner from Gotham, reflects our history for many decades, fighting the false credo of “destroying for saving,” a logic worth the residence in the Arkham Asylum, fitting well the description for a suicidal enterprise.

Batman, born on the eve of the Second World War (1939) and in the aftershock of the Great Depression (1929–1939), became a pop American icon in no time (Goulart, 2001). The success of Batman paved his way toward the screen, where he arrived for the first time “in the 1943 Columbia serial Batman, an odd but fascinating mixture of Batman comic mythos, World War II propaganda and cheaply crafted cliffhanger clichés” (Reinhart, 2013).

In the 1960s, Batman comes back to screen (1966, dir. L. Martinson) for a long career. In every new Batman movie afterwards, there is a growth in the complexity of the characters, and a notable move toward darker atmospheres and more ferocious profiles of his foes. In a way, Batman adapts to the times and audiences as always. However, “neither author, nor medium, nor primary text, nor time period defines the Batman” (Uricchio and Pearson, 1991/2015, p. 208). Thus, the character itself becomes the primary signifier of any Batman narrative. This account also applies to his enemies, like Joker.

In DC comics, Batman first entered just as a sketch of Bob Kane (a riposte to the growing success of Superman). Kane, in drawing Batman, was borrowed from da Vinci's the ornithopter's wings, and the idea of a masked hero, in a sinister looking outfit, like a villain, was from *Zorro and the Shadow* (Goulart, 2001; Reinhart, 2013), as well as from 1920 film *Mark of Zorro* (then Kane was 11 years old kid from Bronx). With *The Case of the Chemical Syndicate*, in Detective Comics #27, May 1939, Batman was born. The story was borrowed by Kane and Bill Finger from Theodore Tinsley's 1936 Shadow pulp story *Partners of Peril* (Reinhart, 2013). Finger patterned his style of writing Batman after the Shadow, “also after Warner Bros. movies, the gangster movies” (Goulart, 2001, p. 88).

In the imaginary place of Gotham City, the Arkham Asylum for criminally insane occupies a privileged place. This imaginary asylum is keeping confined within its expressionist walls all forms of corruption and crime, as well as all folktales' specimens of mad or evil psychiatrists (like Scarecrow/Johnathan Crane or Harleen Quinzel/Harley Queen), some directly inspired by the *Cabinet of doctor Caligary* (1920, Robert Wiene). Arkham Asylum for the criminally insane became, decades later, the stage for a series of computer games (Batman Arkham series, Rocksteady Studios, published by Warner Bros.).

Gotham City is inspired by Fritz Lang's expressionistic *Metropolis* (1927) (Reinhart, 2013; Berland, 2020). Gotham, a city made from many cities, notably in Nolan's trilogy (Chicago, New York, Pittsburg, etc.), resonates subtly with his caped hero, in that “the fluctuating image of Gotham City relates to the fluctuating nature of crime in Batman's world” (Uricchio and Pearson, 1991/2015, p. 187). Gotham itself became a complex text, “a text, that is, a network of different messages depending on different codes and working at different levels of signification” (Eco, 1984, p. 5).

The core character of Batman (Uricchio and Pearson, 1991/2015) grew to achieve several key components, namely traits/attributes (wealth, physical prowess, deductive abilities, and obsession); events (e.g., origin story and fighting crime); recurrent supporting characters (e.g., James Gordon, Alfred, Robin, and Joker); setting (Gotham City); and iconography (costume, colors, cape, bat-like shapes, and bat-prefixed devices that are repositories of the bat-look: black, shiny, with bat-wing design).

In the 1980s, Batman reemerged in The Dark Knight Returns, a story written by Frank Miller, and penned by Frank Miller, Klaus Janson, and Lynn Varley, of a 55-year-old Bruce Wayne/Batman fighting crime after a decade of absence. Here, Batman and Gotham receive a surplus of gritty realism and a darker atmosphere, a move that either Burton's gothic Batman (1989) or Christopher Nolan's Batman trilogy (2005–2012) retained and further developed.

The audiences love Batman, as the hero is always open to new interpretations by different generations (Spiegel and Jenkins, 2015/1991, p. 193), enduring for each generation the needed transformation. The cineasts happen to enlist the caped crusader for many tasks, and, after many years from his debut in 1943, Batman Universe had become a never-ending source of cinematic spin-offs (or television or animated series), where Buce Wayne/Batman happens to be just a reference in the same, yet different Gotham (e.g., 2020, dir. Cathy Yan, Birds of Prey).

As the main character of 11 movies released between 1966 and 2022, within which real turning points were Tim Burton's *Batman* (1989) and *Batman Returns* (1991) and Christopher Nolan's trilogy (*Batman Begins*, 2005, *The Dark Knight*, 2008, and *The Dark Knight Rises*, 2012), Batman grew into an international iconic cinematic character. Moreover, Batman Universe is still developing further. There are a couple of new Batman movies in the making. It should be noted that there are several spin-offs featuring his enemy, the Joker, that got acclaimed for its realism and exquisite composition (e.g., *Joker*, 2019, *Joker Folie a deux*, 2024, dir. Todd Philips), along with various action sagas featuring DC heroes and villains from Gotham. There are two animated Batman series that provided some sources of inspiration for Nolan's trilogy (Reinhart, 2013).

Before moving on with the analysis of the trilogy to identify the elements that may contribute to the activation of depressive dynamics in the viewer, pertaining to the latent content of the films, we will briefly present how Blatt (2004) understands depressive experiences, the perspective we will use in this study. We chose this perspective because it offers an integrative and empirically based conceptualization of depression.

### 3.2 The labyrinth of depression

Depression, from early conceptualizations (Freud, 1917/1957), is seen as a dark shadow cast over oneself. Moreover, loss plays its part. Both classical views and the recent developments, “stress the importance of disturbances in mental representation in the etiology of depression” (Blatt, 2004, p. 49), as well as intense ambivalence with parents in childhood (Abraham, 1911/1927; Freud, 1917/1957), and impaired internalization (Freud, 1917/1957; Blatt, 2004). However, it took a while for us to recognize that feelings of “helplessness; weakness; depletion; being unloved; fears of abandonment; and intense wishes to be soothed and cared for, helped, fed, and protected” (Blatt, 2004, p. 47) are part and sources of depressive states of mind.

In the last decades of research, a unified perspective regarding depression emerged and developed. Blatt's (2004) views regarding depression as anaclitic and introjective are consistent with object relations theories and attachment conceptualizations (Bowlby, 2005), or the theoretical developments from an interpersonal perspective (Arieti and Bemporad, 1978, 1980), as well as the views that emerged from a cognitive behavioral perspective (Beck, 1983). Blatt's understandings regarding depression result directly from the idea that there are, within personality development, “two fundamental tasks: (1) the establishment of stable, enduring, mutually satisfying interpersonal relationships; and (2) the achievement of a differentiated, consolidated, stable, realistic, essentially positive identity” (Blatt and Ford, 1994, p. 6). As such, “The establishment of stable and meaningful interpersonal relatedness defines an ‘anaclitic' developmental line. The development of a consolidated and differentiated identity and self-concept defines an ‘introjective' developmental line” (Blatt and Ford, 1994, p. 7). Both are presenting a dysphoric tone, irritability, losing interest, and eating and sleeping disturbances. Yet, the sources for depressive states of mind are quite different.

#### 3.2.1 Anaclitic depression (dependent)

Blatt (2004) indicates that this line, with narcissistic roots, implies a childlike dependency, manifested in assuming that one is fed, comforted, and soothed, and that the subject has little capacity for tolerating frustration. Subsequently, the experience of being unwanted, unloved, neglected, and abandoned is the constant outcome amplified by a constant reiteration of expectation and failed realization pattern.

Early disruptions of caring relationships, which may include deprivation, inconsistency, or over-indulgence, generate profound anxiety related to fear of loss of love, abandonment, or emotional deprivation. The individual feels powerless to satisfy his or her desire for affection. The separation and loss of the object provoke primitive reactions, such as denial and a frantic search for substitutes.

Stiemerling (2006) notes several common ways adults try to find a way out of anaclitic depression. Initially, there is an attempt to directly address the symbiotic deficit, with their lives focused on seeking a maternal figure. The romantic partner is expected to remedy the lack of happiness experienced thus far and provide the much-longed-for fulfillment. However, this approach is likely to fail, as the demands placed on the partner by the individual suffering from the primary deficit can become overwhelming; this often results in great disappointment, leading to breakups and a repeated cycle of the same dynamics. Additionally, the individual might tirelessly pursue relief from deficit through various substitutes, seeking different forms of satisfaction ranging from food and alcohol to consumerism, an obsession with travel, or spiritual journeys for healing within various sects and groups. Through constant activity, including the enactment of dramatic social conflicts and tragedies in relationships or through workaholism, individuals often attempt to evade the underlying problem.

#### 3.2.2 The introjective depression (self-critical)

This line of depression “is typified by punitive, harsh, relentless feelings of self-doubt, self-criticism, self-loathing, blame, guilt, and depression” (Blatt, 2004, p. 48). Consequently, hardly compensating for feelings of inferiority, shame and guilt are common. Standards are high, and a constant drive to perform and achieve is present. They are correlated with guilt and shame for not living up to expectations, reflecting the internalization of the critical values and attitudes of authoritarian parental figures. A strong sense of morality and intense self-involvement lead to constant self-evaluation and are fundamental expressions of depression. The feeling of guilt arises as a result of temptations and transgressive thoughts, often related to the Oedipal rivalry theme, and from the individual's belief that he or she does not live up to expectations and standards, leading to disapproval and criticism.

The pressure to be perfect, the tendency to take responsibility and blame, along with the feeling of inability to gain approval, acceptance, and recognition, contribute to the development of depression. Extreme standards, often over-valued and attributed to external figures, coupled with intense preoccupations with disapproval and punishment, generate ambivalent and hostile feelings toward others. Depression develops out of deeply ambivalent, demanding, and dismissive parent—child relationships, manifesting in severe self-criticism, self-hatred, self-blame, guilt, and intense engagement in activities aimed at compensating for feelings of inferiority and worthlessness. Efforts by the individual to gain approval and compensate for feelings of inadequacy are so intense that they affect their ability to enjoy life.

Blatt (2004) mentions that the two forms of depression are not mutually exclusive. The most severe form of clinical depression seems to result from a combination of dependency and self-criticism. This interplay between the two sources of vulnerability can generate extremely intense levels of depression, creating a unique and very difficult situation to manage.

Experiences of depression are not found in clinical settings alone. Blatt talks about a continuum ranging from normal to severe clinical states. Early experiences may predispose some individuals to be particularly vulnerable to experiences of abandonment and loss or experiences of failure and worthlessness. These vulnerabilities may be the consequence of representations of others as untrustworthy and uncaring or as intrusive, judgmental, and punitive, and of representations of self as unlovable and undeserving.

In the following part, we focus on the movies to identify the latent contents that are recognized implicitly by viewers. We can get to these by analyzing the film (as manifest content). In the systematic analysis of the film, we take into account Mikos's (2013) five dimensions: content and representation, narration and dramaturgy, characters and actors, aesthetics and configuration, and context.

### 3.3 Why do we fall Bruce?

We find Bruce Wayne at age 28, somewhere in a prison in Bhutan, surviving in miserable conditions. He is still tortured by nightmares of bats, studying criminals and learning to fight and defeat them, crazed with anger, and strangled by guilt over the loss of his parents. We know he has been feeling guilty since the night of the murder, feeling that he is to blame for their deaths, that if he had not been afraid and did not want to leave the opera early, they would still be alive. It seems that Bruce is trapped in pathological grief, from which he cannot get out, thus turning into depression. An introjective depression, marked by feelings of guilt, of self-punishment, in which he isolates himself, retreating to the end of the world, to a place where he is alienated from others, is different. He chooses to put himself on guard, to defend from the aggression coming from outside, thus avoiding looking at the nightmare from within. Moreover, he is highly ambivalent about himself. If we look at the situations he stages for himself, we could say that he hates himself and puts himself in contexts where he has a lot to endure—hunger, deprivation, and humiliation. We also see a trace of self-love manifested vicariously, from distance, by funding an orphanage where he gives support to children who have lost their parents to violence, perhaps recognizing himself in them and protecting himself by identifying with them.

Why this failure of mourning? Why has the normal grieving process turned into depression? In describing the mourning process, Bowlby (2005) identified a sequence of responses to the loss: protest, despair, and detachment. Following unexpected loss, there seems always to be “a phase of protest during which the bereaved person is striving, either in actuality or in thought and feeling, to recover the lost person and is reproaching him for desertion” (Bowlby, 2005, p. 62). During this phase, emotions can be conflicting. The individual's mood and actions may swing from a hopeful yet angry demand for the return of the lost loved one to a deep, quiet yearning that may sometimes go unvoiced. “Though alternating hope and despair may continue for a long time, at length there develops some measure of emotional detachment from the person lost. After having undergone disorganization in the phase of despair, behavior in this phase becomes reorganized on the basis of the person's permanent absence” (Bowlby, 2005, p. 62–63).

Typically, the blockage occurs when the child is unable to process the loss because there has not been someone at his side to provide a supportive, empathic mirroring, to offer maternal love and support in going through the process. We see Alfred offering Bruce empathic mirroring of his longing for his parents and support in dealing with his guilt. He acts as a maternal substitute—he is the one who nurtures and cares for the child. However, he does not seem to be able to sustain Bruce to get through the other phases of grief, disorganization and despair, to be followed by reorganization. In a way, the trilogy illustrates the journey of Bruce's coming out of depression and going through the process of mourning, in the end becoming able to love, to accept to be loved, and to establish affectional bonds.

This blockage could be an indicator that it is not only the longing that is troubling Bruce. The death of the loved object may also fulfill unconscious death wishes for the departed. Was the young Bruce jealous of his parents? Did he feel excluded from their love, symbolized by the pearl necklace his father had given to his mother? Did he envy them for being happy without him? (Britton et al., 1989). Did he enter a downward spiral, encompassing feelings of guilt, a dull awareness of one's own bad character, leading to self-punishing tendencies?

Behind the longing could also be hidden anger toward the parents who, through death, have abandoned him? Anger at the father, who failed to protect his family? Anger at himself, who was too small, too vulnerable, too powerless to fight the murderer? Perhaps the meaning of prison fights is precisely this—to try and succeed in defeating the offender, re-enacting, with every conflict, the scene in the alley, to escape the shame awakened by the narcissistic wound of helplessness and passivity. Bruce does not want revenge; he wants to be able to face the enemy. To this end, he invests all his resources—physical, mental, and financial.

It would seem that beneath the introjective aspect of the depressive experience that appears in the foreground, we can also distinguish the anaclitic one—as an orphan, Bruce is in a state of emotional deficit, the abandonment can be experienced as a narcissistic injury, as if he did not deserve to be protected, he lost the model of identification that he had in his father, and at the same time he also experienced a disappointment related to his father's weakness and cowardice.

### 3.4 Who is Bruce Wayne and how he and his world are shown to us by filmmakers?

One can say that Bruce is a narrative form, endowed with a complex inner world by his many authors (Christian Bale, Christopher Nolan, Jonathan Nolan, David Goyer, Wally Pfister, and Nathan Crowley) and “existing” in a complex projective narrative visual field.

All the masked characters, living their “lives” in Gotham, could be redistributed within Bruce's inner Gotham, identifying in this new casting of roles of both the “dreamer who dreams the dream” and the cinematic interpretations of his turmoil, of his inner suffering enacted within and through the actions of his enemies. One short description of Bruce's journey could be: he frees himself from deception and guilt, i.e., from depression, to live his own life.

In terms of narrative and dramaturgy (Christopher Nolan, Jonathan Nolan, and David Goyer), there are few instances when the audience is led by the movie to believe something that later was revealed as false. The identity of Rā's al Ghul is one example (*Batman Begins*), and the identity of his child, Miranda/Talia, is another (*Dark Knight Rises*). In both cases, a decoy is employed, installed by the deceiver, “presenting” himself or herself as an ideal form—a narcissistic trap, or an illusion.

In terms of cinematography (Wally Pfister) and editing (Lee Smith), as well as in relation of the changes seen in set design (Nathan Crowley), there is a subtle move from a realistic presentation of the “world,” one endowed with a high degree of plausibility, toward a sophisticated nightmare resembling sequence of shots and sets, enhanced with CGI, filled with smoke and mist. This filmic, subtle transition from real to oneiric, into which we are drawn, shows on screen, at image level, a process of deteriorating contact with what is “real,” within this slow move from a “real” to a “post-apocalyptic like” Gotham.

In terms of editing, set design, and cinematography, there is a short episode—Bruce is tested by Rā's al Ghul—that follows the previously drafted shape, from “real” to “oneiric” depiction of the world. The scene gradually reaches a maximum conversion of the “real” world into an “oneiric” one, by following Bruce's levels of intoxication and fear, after breathing in the smoke of the blue flower filled with a hallucinogenic substance.

In *Batman Begins* (2005), we found the same game of “smoke and mirrors.” Bruce gets his training in “deception and theatricality.” Training unfolds as Bruce is subjected to “deception and theatricality,” e.g., the attempt of Ducard to brainwash Bruce's father from Bruce's mind or duping him to believe that “justice is revenge.”

Nevertheless, we are also “deceived,” regarding who is who and what purpose follows. The movie lets us experience the way out of deception, as we become subjected to disillusionment and subsequent realizations. It creates depth to the dream world of the silver screen we are engulfed in. One's contact with what is “real” (or not) gets juxtaposed with a subtle fact, unknowingly passing by most of the time. Our wishes and our perceptions are not as disjunct as we would like to believe. Freud realized more than a 100 years ago (e.g., Freud, 1905/1953) that they modulate each other to “partially coincide” (Ruesch and Bateson, 1951, p. 200; Mikos, 2013, p. 156). As such, “perceiving” fictional worlds is modulated by wishes and fears evoked by the narratives unfolding in front of our eyes. Explicitly, the above sequence could be described as having an ideal (e.g., Ducard), losing it, and escaping deception by recovering a good object (Bruce creates Batman, and subsequently regains his father's name and legacy). The ideal is transferred into his mask, into Batman. Evading the prison house of the narcissistic states of mind, revenge or shame, induced and cultivated, implies recognizing what and how something or someone really is.

Eventually, in *Batman Begins*, the induced fear followed by hallucinations can be faced without losing one's mind. So, one is self-contained and able “to perceive” what is real (e.g., Rachel or Gordon), under the works of an “antidote.” A valuable aid, obtained following the pains of the “chemical induced fear,” just after Bruce/Batman got defeated by Scarecrow's cocktail of fear. A divorce from reality that Bruce had experienced earlier, in his training in “deception and theatricality.” These tools, manipulating and dividing one from his own self through brutally induced fear and confusion, are mirrored in how we appropriate these narrative movements presented on screen. We move from being “deceived” to seeing what is going on, as there is an antidote. In experiencing what is happening in the “now” of the film event, we transform the story of Bruce, time and time again. As an emotional and cognitive subjective sequence of events, there is also an acting on our own fears, needs, and wishes stirred up by the film.

“We think in stories” (Bateson, 1979, p. 14). The cinematic games of depictions, interpreted as “real” and “oneiric” strata of a narrative world, have retained in their subtle dance the ways employed by Batman's primary sources—the paper worlds of comics, built within the minds of their readers. About these worlds, Umberto Eco, in his essay on Superman, says that “the stories develop in a kind of oneiric climate—of which the reader is not aware at all—where what has happened before and what has happened after appears extremely hazy” (Eco, 1984, p. 114). This oneiric climate can be recognized also in Nolan's “realistic” construction of the trilogy and the way we appropriate it, as sketched above.

Below these levels of intertextuality and transferences, there is the depiction of the fight with depression.

### 3.5 And yet, who is Bruce Wayne?

“Who is Batman?”—Inside the fictional world of Gotham, this is the question asked by so many. The audience knows the formal answer—Bruce Wayne. Who are Rā's al Ghul, Bane, and Joker for him? Are they facets of Bruce Wayne, a kid tormented by the violence of loss, grown up into a man? Are they becoming the personifications or masks of an inner violent struggle of a depressed little boy surpassing an impossible task? Batman is born from loss and grief. All fans know that. So, one can say that he needs his villains, all those gangsters, terrorists, and murderers. Moreover, Gotham, as a symbolic representation of Bruce's personality, needs Batman. As such, we can ask: Are Batman, Joker, and Bane representations of Bruce Wayne's various depressive experiences?

#### 3.5.1 Batman

All Bruce's depressive states of mind are emerging as masks fitting either the “preoccupations with the object's wellbeing” or “with the subject's self-worth.”

Batman's mask and cape have an intricate history. In Batman Begins (2005), we witness, just after the scene of brutal shooting of Bruce's parents, the tenderness and recognition that Gordon manifests, while facing the pain of little Bruce Wayne. Bruce is sitting in the captain's office, “bewildered and forgotten,” clutching his father's coat. Moreover, Gordon drapes him gently in his father's coat. The gunman is caught, and now he is in police custody. Gordon's paternal gesture is a metaphor for the overall mask, the well-known black cape made from “memory cloth,” making visible what identifications are at work. The silhouette of Batman, his well-known cape, relates to his father's overcoat and legacy suffused with loss and revenge. The father's credo, to cure people's illnesses, is transformed into “curing city's illnesses” for Bruce. Then, it was too big to fit a little boy, so the depth and pain of his loss got preserved within the draped shape of the Black Knight, as a mark for a “constant anger that outweighs his guilt.” His most profound feeling in the beginning is anger, “directed in the first place against the self, for the false belief that it could escape its depressive fate. It is directed against the world of others, who are experienced as having failed the self” (Bollas and Bollas, 2021, p. 56). Bruce is a subject under the “unsatisfiable cathexis of longing” (Freud, 1926/1953, p. 172), and his feelings are fixed deep within him, fueling his identifications and building up a mask for them, for perpetuating this constant, “non-movable anger that outweighs his guilt.” He is wearing the mask, night after night, while fighting to reestablish law and order, being the cure of a sick city. Beneath his armor, he is under the illusion of protection; this identification protects also the little boy inside him. He is upright and principled, and, at the same time, his mask helps him to avoid mourning, even if this means he embraces depression and loneliness.

#### 3.5.2 Joker

His mask has different stories, while he has none. Nobody knows who he is or where he is from. He has no past, except our recollections of him. However, Joker delivers several stories about his mask. First instance occurs with Gambol, one of the Mafia lords, another with Rachel, and the third one, an unfinished story this time, with Batman. He begins inviting: “Wanna know how I got these scars?”. Both stories are tales about how one's soul arrives to be depleted by the loss of the needed one.

In the first story, his mother is hit by his father, who turns to him and says “Why so serious?”. The second loss: she is a gambler, and got her face carved by the “sharks,” and Joker says: “I just want her to know I don't care about the scars. So, I put a razor in my mouth and do this to myself … And you know what? (Starts laughing.) She can't stand the sight of me … (Or crying.) She leaves!” (Nolan et al., 2012).

Joker is a “freak,” “simultaneously and compulsively fascinating and repulsive, enticing and sickening” (Grosz, 2020, p. 274), and his face is his mask (in the one second paintless face close up, from *The Dark Knight*, appearing in the dispersion of the shooters after the failed assassination, one easily recognizes Joker, so his mask is made from scars and smirks). What Joker delivers in *The Dark Knight* (2008) is an anti-ideal, an anarchic, sadistic object, the abject maker (Kristeva, 2020).

Joker is not only a freak, or a chaos maker, he is a deject, a “situationist in a sense, and not without laughter—since laughing is a way of placing or displacing abjection” (Kristeva, 2020, p. 100). Kristeva's description of the deject, the one by whom the abject exists, seems to illustrate quite well Joker's face work: “he divides, excludes, and without, properly speaking, wishing to know his abjections is not at all unaware of them. Often, moreover, he includes himself among them, thus casting within himself the scalpel that carries out his separations” (Kristeva, 2020, p. 100). Yet, for all these to happen, jealousy and envy performed within and onto oneself are needed (Searles, 1986).

Joker appears as Bruce's internal chaos maker apparatus and can be seen as his envious object, his “deject” nemesis, instilling the subtle play back of trauma, repeatedly. Joker can be seen as a self-destructive part of Bruce. As such, “he” participates in how Bruce is hurting himself unknowingly. If Joker is seen as a part of Bruce, he represents an internal object unfolding an envious attack. This envy “is a major factor in maintaining the disharmony” (Searles, 1986, p. 100) of one's inner world. The actual chaos engulfing Bruce's world is the result of a splitting. If Joker is seen as an envious part and Batman a loving one, love must suffer irreparable damage under envy, leading to self-retraumatization.

Depression can be defined by Joker's line (while he gets defeated by Batman): “this is what happens when an unstoppable force meets an immovable object.” If we listen to the stories of Joker, then we meet another traumatized child. However, in his case, the mask is irreparably imprinted on his face by his father or by himself. He smiles all the impossible smiles (“See, now I see the funny side. Now I'm always smiling”) because others (the father and the wife) could not bear his seriousness and concern. He is denied the expression of love, and he is left only with hatred and envy. The emotional scars of violence remain forever, in a prohibition of sadness and care for the other. It is a mask he cannot remove, a characteristic he cannot change, deeply imprinted in his identity. He is his scar. A scar of narcissistic injury.

#### 3.5.3 Bane

Bane lives through his mask; his mask alleviates suffering. Without it, life would be far too painful. Thus, his mask becomes a sign for trauma. We learn that Bane comes from an ancient cave where people are thrown to suffer and die. It is a cave where Miranda/Talia's mother was thrown, and here she gave birth to Miranda/Talia. In this “prison,” Bane became her protector. An extremist, he was excommunicated from the League of Shadows, considered a monster that can never be tamed.

Bane seems to be a “murdered soul” (Shengold, 2011). Soul murder “is a crime in which the perpetrator is able to destroy the victim's capacity for feeling joy and love” (Shengold, 2011, p. 121), so the idea of Hell, of the very dark pit of nothing but trauma, of the life lived having “to bear the unbearable, alone, forsaken by parents and God” is fueled by nothing but hope, so nothing good arrives to survive.

The pit can be seen as the narcissistic wound that will never heal, which, from time to time, sends to the surface, to the self, a new depressive episode. Being Talia's birthplace, the pit is a source of her and Bane's relationship; the pit is linked to a lost mother. Through Bane and by her own works, Talia becomes the image of depression and cruel punishment, in the silent and sly act of vengeance of a lost father. In her triumph over Batman, while torturing him, Talia gives words to her depressive state of mind, her own Hell: “You see, it's the slow knife … the knife that takes its time, the knife that waits years without forgetting, then slips quietly between the bones … that's the knife that cuts deepest” (Nolan et. al., 2012).

In Bane's new worlds of Gotham's sewers and blackness, there are so many unwanted children forgotten into the black pit of nothingness. Bane is the ruthless dictator of the army of abandoned and unwanted children. The story says that he got ill in the pit, and a medical error endowed him with his mask—a respiratory prosthesis, a filter for pain. He is the living failure of those who deliver care. He resembles the child of the mentally dead mother (Green, 2001), a child imprisoned in depression, overexposed, and lacking the mother as a filter of overstimulation.

### 3.6 Why do we fall, Bruce? So, we can learn to pick ourselves up!

From a psychoanalytic perspective, regarding models of depressive states of mind (e.g., Bleichmar, 1996; Blatt, 2004), there are several paths of reading the Batman trilogy. However, these depressive states of mind are polarizing mental pain into a pair of very different ways of dealing with loss and depressive anxiety. Moreover, for Bruce, all began with loss and guilt, with isolation and anger.

#### 3.6.1 Batman Begins (2005)—The struggle with guilt feelings

We can discern in the creation of Batman a fracture in the ego, a process that Freud describes in “Mourning and Melancholia” (1917), when the ego splits, cleaves into two parts. One part carries the phantasy of the lost object. For Bruce, this takes the shape (and aims) of the bat mask, armor, and cape, carriers of his identification with his father (who carefully dresses his wounds made by fear and who lifts him up from the pit filled with bats). The other part becomes a critical agency, the shadow “fallen upon the ego” (Freud, 1917/1957, p. 249). Moreover, the Shadow becomes the black specter of justice, haunting Gotham villains. Not so different from his predecessor, Hamlet, Bruce undergoes a major transformation—through his mask, the bat, he becomes a “specter” that cures the city. “The specter is not simply this visible invisible that I can see, it is someone who watches or concerns me without any possible reciprocity, and who therefore makes the law when I am blind, blind by situation. The specter enjoys the right of absolute inspection. He is the right of inspection itself” (Derrida and Stiegler, 2013, p. 41). In *Batman Begins*, we witness how the journey out of depression sets its course, with the creation of Batman and the bat mask as recognition of his identification with his father and his ongoing fight for being free from it (Brill, 1983).

Bruce meets his first enemy. As “teacher in deception,” Rā's al Ghul qualifies from the very beginning as an anti-father, a deceptive ideal. Thus, Bruce meets deception, the employing of illusion that breeds fear. Why is Rā's al Ghul offer so tempting? Bruce was experiencing the deep bites of depression, in a miscarried repair of his injured self. A drifter, running away from his inner pains, finds out, like all of us, that nowhere is granted relief from his own ghosts. Bruce, far away from Gotham, presents “a profound sense of loss of agency and a sort of sacrificial capitulation to depression as a punishment of the self” (Bollas and Bollas, 2021, p. 45).

He discovers the bitterness of the failure of his anger, so he is facing the feelings of impotence (Bleichmar, 2010, p. 205) and gets involved in activities of training to survive. This was not misrecognized by Rā's al Ghul. The deceptive father figure cannot miss the punitive, harsh self-criticism of Bruce, his self-loathing, blame, and guilt, as well as his “intense involvement in activities,” designed to “compensate for feelings of inferiority, worthlessness, and guilt” (Blatt, 2004, p. 32). Step by step, the trade offer of changing anger for revenge, love for hate, is made. In the end, it looks like: Gotham should vanish, which would be a “suicide” for Bruce.

Yet, Rā's al Ghul misses that his offer of trading depression for disguised psychopathy cannot fit Bruce. Losing his parents to death is one thing; losing their love is another. Self-deception equates to gaining a way out for the price of a double loss. Trading a good inner father for a deceptive one, as the offer goes, is equivalent to trading anger and guilt for rage and shame, i.e., losing his good object again, this time from within. It can be said that Rā's al Ghul forced an identification of Bruce with his father, making him a protector, a future defender of Gotham and of his own self.

As a mentor, Rā's al Ghul also offers a father figure for Bruce to fight with, a man who could face his rage, who could survive the blows, much needed because it is impossible to fight someone “*in absentia* or *in effigie*” (Freud, 1912/1953, p. 108). The scene where they wrestle on the ice while having a dialog about Bruce feeling guilty for nothing, his father was guilty for not defending them, resembles a psychotherapy sequence, where truths about the past are told. They change the way Bruce sees himself, and emotions hitherto locked are expressed in a relationship with a paternal transference figure. On the frozen battlefield, feelings “defrost,” anger is manifested, disappointment is also present, and the burden of guilt is lifted. A catalyst for mourning, as now Bruce can be in contact with his ambivalent feelings over the loss of his parents, without being overwhelmed by guilt over his aggressiveness and by the fear of punishment. Moreover, what Bruce achieves is recognition from Gotham, and the capacity to repair.

#### 3.6.2 The Dark Knight (2008)—The struggle with envy and resentment

*The Dark Knight* defines Batman and Joker as complementing each other. They cannot live one without the other, and this is emphasized by the scene where outside the mayor's office, a dead body wearing both masks (of Joker and Batman) hangs in front of the windows. A secret message that only Bruce can understand.

The duality is at the center of the movie, where we meet different pairs: the Black Knight and the White Knight, Batman and the Joker, and corrupt and honest policemen. Moreover, the world seems to be split in two parts, with aggressors and victims, poor and rich people, culminating in the end with two ships, one with innocent people, another one with criminals, Joker who wants to burn only half of a pile of money, the lucky coin which flips in the air, with identical sides. It seems that the Gotham world is ruled by splitting—a splitting between good and evil parts, creating a playground dominated by Mafia, the only one who can negotiate and make alliances with one part or another. Symbolically, Gotham's situation indicates a split in Bruce's personality into good and bad parts, and the presence of an inner Mafia, which has the purpose of maintaining the status quo and the power of destructiveness, similar to the inner situation of the patients described by Rosenfeld (2004). In these patients with destructive narcissistic organization, envy is usually more violent and more difficult to face, and their progress is often accompanied by the appearance of violent self-destructive impulses. Such patients often act in self-destructive ways by spoiling their professional success and personal relationships. “Some of them become very depressed and suicidal, and the desire to die, to disappear into oblivion, is expressed openly. Death is idealized as a solution to all problems” (Rosenfeld, 2004, p. 107). Rosenfeld notes that frequently, when a patient of this kind makes progress in understanding himself and wants to change, he dreams of being attacked by members of the Mafia, representing a narcissistic organization of the self to maintain the idealization and superior power of destruction, and defensively keeping itself in power and so maintaining the current situation. “To change, to receive help, implies weakness and is experienced as wrong or as a failure by the destructive narcissistic organization which provides the patient with his sense of superiority”(Rosenfeld, 2004, p. 112).

The movie begins with a robbery, and many of the villains are thieves. The Mafia is doing illegal business, stealing money, and selling drugs. It seems that the main conflict is given by a fantasy of robbery, in which the good is robbed by the bad, and what seemed to be a splitting between good and evil becomes a splitting between poverty and wealth, between having and not having, representations accompanied by corresponding affective charge—envy and resentment of those who do not have over those who have. In the film, Joker is similar to the “resentful man” described by (Scheler (2010)), who no longer seeks to denigrate and destroy a single envied object. However, his envy has already transformed, and destructiveness manifests itself on an impersonal level, generalized in relation to an entire class of individuals, a man who destroys for the sake of destruction, not for a personal revenge.

If the Joker is also a part of Bruce Wayne, then Bruce Wayne, the prince of Gotham, is also consumed by envy. Given that he is the richest man in Gotham, his deficit cannot be financial. The Joker, marked forever by his narcissistic deficit, has the answer.

We do not know too many details about Joker's life. However, we get the gist, recollections, or fictions about—he had a violent father who mistreated his mother and him and, presumably, a mother who rejected him, if we understand rejection from his wife as an echo of his relationship with his mother. Children brought up in such families, we learn from Celani (1994), are children who carry two burdens—on the one hand, they feel abandoned by their father because, instead of protecting them, he hurts them, and by their mother because she is powerless to stop the violence. They also feel ashamed of not being loved, believing that they do not deserve it as they are not worth enough. In this context, we discover the Joker's narcissistic wound—his belief that he cannot be loved and its follow-up: envy of all the other people who have received more love than he has.

If Batman represents Bruce's identification with the idealized father, does the Joker represent the part of Bruce disillusioned with his father? The sudden loss of the father may have awakened in the child not only a sense of having been abandoned through death, but also a sudden, traumatic desidealization (Kohut, 1984). The all-powerful father, with whom he had previously felt safe and proud, suddenly turns out to be fragile, vulnerable, unable to protect his family, a coward, as Rā's al Ghul claims. This sudden desidealization can simultaneously lead to a sudden collapse of the son's self-esteem, who can no longer lean on the father's strength to feel strong, and to feelings of shame about the father's weakness and cowardice. It could be experienced as a narcissistic injury to his self-image as he becomes acutely aware of his helplessness, of his limitations, of the recognition that participation in parental omnipotence was an illusion—the marks of narcissistic depression.

On the other hand, Bruce's mother also seems to be absent in the relationship with her son, as she accepts far too easily the husband's lie, who, trying to protect the son from the discomfort of being considered a coward, claims that he wanted to leave the show. Fromm (2013) observes how painful it is for children to succeed in lying to their parents. The implications are two-fold—on the one hand, children are left with the feeling that their parents do not know them well enough or are, in fact, indifferent to what is happening to them, “too disinterested to know better” (Fromm, 2013, p. 65). Therefore, whether she believed the lie or did not pay attention to it, Bruce's mother appears to be indifferent to her son.

Thus, Joker appears to represent that part of Bruce that is narcissistically injured by the lack of love from his mother and lack of protection from his father, a part filled with shame, humiliation, envy, and resentment.

#### 3.6.3 The Dark Knight Rises (2012)—Struggling with self-hatred

At the beginning of the third part, Bruce is depressed, “self is still there, but it exists now as the ghost within” (Bollas and Bollas, 2021 p. 60). Moreover, this ghost within is the lost Batman, the doer, the punisher of the villains. With Bruce's alter-ego gone, nothing else moves anymore. So, “the relation to this ghost is a profound secret. At times it seems as if the cloak of depression is meant to hide it, to keep it out of sight of the observing other” (Bollas and Bollas, 2021, p. 60). Besides losing Rachel, Bruce became part of an untruth that implied the exile of Batman. In the line of his duty, of justice and responsibility, Bruce's pain follows his identification with his ghosts. So, he confines himself within a personal madhouse. There is no armor and cape, but a veil of depression. There is no Batman to contain his ideal father by healing the city, and no other outlaw but himself. He is experiencing a double loss, for which, again, he is to blame. So, Bruce endures years of grief. An invisible exile, of 8 years long, matching the age of losing his parents. Bruce's personal Specter, the absence of Batman, points to the underlying compromise, which “indicates that, beneath the surface of received history, there lurks another narrative, an untold story that calls into question the veracity of the authorized version of events” (Weinstock, 2013, p. 63). This untold story, as Bane proves, is the story of Bruce's hatred. Bane can be seen as another face of Bruce's depression. The unseen side of Bruce journeyed from guilt, deception, and fear, toward envy and abject, to arrive at its core, that is hatred and violence, the roots of all evil.

Exiled, Bruce is making more room for falling-off. Self-hatred is now visible. And his hate, much bigger than ever, is contained within isolating himself in the east wing of Wayne manor, in his personal Arkham. The works of hate in this self-exile, make visible the feelings of abandonment, of being unwanted, of being guilty for all of that. So, before Bane, Bruce experiences blame and punishment.

Bruce shows a first sign of vitality when he gets robbed, and that happens when his pact with Gordon is reiterated—the Harvey Dent commemoration day at Wayne Manor. In a subtle link, Bruce interacts with Selina through an arrow. Like in the beginning, when playing with Rachel. Then, Bruce steals from Rachel the “finders keepers” stone arrowhead, followed by his falling into the bat pit. She steals (his fingerprints and mother's pearls) and jumps out of the window.

So, we find an exiled Bruce, like Bane. Moreover, crippled by depression, thus nothing like Bane. Exile completes the link between Bane and Batman, made also from the feelings of being unwanted and abandoned. Moreover, Bane makes his move toward Gotham. It looks like Batman's last nemesis is summoned by Bruce's years-long kept pact, made with Gordon, by the paradox of enacting an untruth for the triumph of law. And in this path, Bruce learns that “the law and mourning have the same birthplace, that is to say, death” (Derrida and Stiegler, 2013, p. 48).

With the arrival of Bane, the city turns upside down. What is good and right goes below, and hatred emerges, engulfing the city. Batman opposes this new hatred-driven enemy and gets defeated. Why? If Bane is seen as part of Bruce, one filled with murderous hatred, then their confrontation “beneath the surface of the city” mirrors the level of conflict and magnitude as would be experienced by a victim of “soul murder,” as an unwanted child dumped at the limits of psychical survival. It is as their fight acknowledges that love is not there; thus, mental health is not there, but a legacy of hatred, of a crippling hate.

“Love must be felt in order to acknowledge and attenuate the terrible legacy of murderous hatred that has become too much to bear without destructive or self-destructive action accompanied by crippling inhibitions and defenses.” (Shengold, 1989, p. 322)

Bruce needs all his resources of love, past and present, and that amount of love is not there; only his duty remains. The single outcome is that Bane (depression) seizes power, and Bruce gets dumped into the very pit from where Bane emerged. Bruce is broken and sunk further down, where hope is the main poison of the mind. For a “murdered soul,” feeling love is felt baneful. Same for Gotham and Gotham sewers. Their champions trade places; Bane colonizes the city, like an internal Mafia seizing power over one's mind, isolating the city to destroy it, as Bruce is sunk into the pit of despair for being wasted.

In the pit, broken, Bruce gets help from a consumed prison doctor, a father figure, about whom we learn that his medical error gave Bane his mask. The doctor and his friend are teaching Bruce the wish to survive, like enacting the old answer to his father's question: “And why do we fall?”. Bruce learns “how to pick himself up,” how to make the leap by facing his fear and not embracing oblivion or the rope of dependence. Contrary to the fight with Bane, he makes the jump free, but being free means that he is capable of love and being loved.

Gotham becomes a large prison house and an asylum as well, an isolated island where the judge is the psychiatrist specialized in inducing fear, Scarecrow, now set on a high level, offering “justice” to the “guilty” through a double bind kind of choice: death or exile, while exile equates death.

Gotham is threatened by an imminent catastrophe, a danger of annihilation; the world can disappear at any moment. Moreover, that is because Bane, the unwanted, abandoned, rejected child, left to fend for himself in the cold, inhospitable world underground, is just a hateful monster, because that is all he is ever gotten from his parents, from the world. He represents the deepest layer of depression, the psychotic level, which can swallow everything in a self-destructive gesture. “People who have been traumatically abused are saddled with the worst expectations-terrifying anxiety, loss of control, feeling like killing and being killed” (Shengold, 2008, p. 289). As with the victims of “soul murder” that Shengold describes, the onslaught of hatred must emerge. In confronting Bane, Bruce is fighting for his mental health. Batman manages to avoid catastrophic annihilation, and for saving Gotham, arrives at making his ultimate leap—a sign that the present episode is over. Moreover, Bruce emerges from depression being able to love and to accept love. However, the scars remain, the pit is still there, and it may send to the surface a new depressive episode. For that moment, there is Robin in the cave, taking over Batman's legacy.

Batman's statue from the end of the trilogy can be read as gratitude. Either for his sacrifice for Gotham, or for leading one out of depression.

## 4 Findings/observations

The systematic analysis of the film revealed aspects of latent content related to different experiences of depression, a process of mourning, and the resumption of development blocked by loss. The trilogy offers numerous opportunities for identification for the viewer, providing the opportunity to retrace an emotional journey. Each viewer will create their own Batman at the intersection of these elements and their own personality. “Cinema is not therapy, yet every great film touches us in some way as if it reminded us of something” (Hamburger, 2024 p. 227).

### 4.1 An instance of triangulation

Berman (1997) identifies two types of (counter)transferential currents, both influencing the viewer's experience when encountering the film universe. “The spectator's response may be experienced mostly as an analyst's countertransference, when figures, works of art, or artists are primarily viewed as enigmatic, as needing to be explained, and at the extreme end as being pathological. Alternately, the reader's or spectator's emotional set may be closer to an analysand's transference, when the work, its protagonists or its creator are primarily experienced as valuable and a source of insight” (Berman, 1997, p. 984).

The analysis we have done so far is colored by our countertransference to the film and by the way we are used to working with the emotions aroused in us by the depressive states of our analysands in our daily practice (see also Edwards, 2010 for the relation between countertransference and the experience of film viewing). However, we also turned to the film as a source of growth and insight, using the dialogs between us and the group discussion for triangulation. Moreover, the trilogy has also become a source of self-understanding.

One of us (S.R.) discussed Batman Begins with a group of psychotherapists and MA students as part of a seminar on Applied Psychoanalysis. Usually, in such groups, we watch the film together (or read a fairy tale), followed by a discussion. My role is to guide the discussions, linking the ideas that arise with different threads of interpretation that emerge, or with different psychoanalytic theories, or proposing new directions for interpretation based on an emotion that seems to have no sufficiently good explanation. Usually, at the end of these meetings, we reach an agreement between the meanings discovered and the sequence of emotions experienced during the viewing of the film.

However, since Batman seminar was an online meeting, participants watched the film the day before so that the details and emotions evoked by it would be fresh in their minds. At the end of the meeting, I stated that “nevertheless, it seemed to me that the film illustrates stagnation, and Batman does not change, he is stuck in a depressive state of mind.” The students' reaction was one of disappointment. I ended the seminar with a feeling of sadness, somehow regretting that I had chosen Batman for discussion. However, I realized I was making a mistake. In my personal viewing of the film colored by my own psychic reality, I considered Batman and Bruce Wayne to be one and the same character. Although Batman remains the same, Bruce begins a slow process of change. This was a significant mistake for me, revealing my identification with a Batman-like protective object, deeply rooted in my personal history, which had a major effect on my choice of a helping profession. Stepping out of this identification, the film began to take on new meanings.

## 5 Discussion

From his inception, “*The Case of the Chemical Syndicate*,” in Detective Comics #27, May 1939, Batman has been a container for hope and uprightness. Is Christopher Nolan's Batman trilogy (2005–2012) a depiction of the struggle with and the process of overcoming depression?

If we center our view on Batman only, and keep Bruce Wayne as an alter ego for him, then it is hard to support such an idea. However, if we revert it to consider the evolution of Bruce within the trilogy, of what he endures and of what he is overcoming, then the idea is well reinforced by the cinematic development of the main character. Considering Batman and the masked villains (Scarecrow, Joker, Bane, etc.) as those conflictual parts of Bruce, then outlining Bruce's emotional journey from the beginning to the end, we will encounter the following: The first conflict (*Batman Begins*, 2005) brings in the foreground the overcoming of fear and guilt, of deception and confusion played out not only between Batman and his foes, but also between different other characters of the plot. Guilt, self-deception, and fear of punishment are prerogatives of a depressive state of mind, and after the first part of the journey, they are changed for recognition of his own value and strengths. In the second part of the journey (*The Black Knight*, 2008), we encounter the overcoming of envy. In sacrificing oneself for others' wellbeing, Bruce comes to accept the feelings of gratitude, disabling the power of shame. In the end (*The Black Knight Rises*, 2012), Bruce overcomes hatred and becomes able to love again, and thus to be free. Restoring within himself the self-respect given by recognition, self-appreciation given by accepting others gratitude, and the ability to love and accept love are all preconditions of overcoming depression and detachment, restoring within oneself the capacity to form again affective bonds.

As spectators, walking in Bruce's footsteps, we embark on this journey, and along with Batman, Joker, Bane, and all the others, we bring to life something of our own depressive states, we go through a mourning that we had to work through, and we strengthen the hope that, finally, there is someone there, someone we can rely on, someone who can protect us, who can accept us as we are—with our fears, with our scars, and with our fragilities and strengths.

Christopher Nolan's Batman trilogy set Batman again in the hearts of many. Perhaps this is due not only to the quality of the films, which are wonderfully written, beautifully shot and greatly interpreted, but also to our deep need for hope, order, and beauty.

## Data Availability

The original contributions presented in the study are included in the article/supplementary material, further inquiries can be directed to the corresponding author.
